# A single high‐fat meal alters human soluble RAGE profiles and PBMC RAGE expression with no effect of prior aerobic exercise

**DOI:** 10.14814/phy2.13811

**Published:** 2018-07-25

**Authors:** Kelly N.Z. Fuller, Rudy J. Valentine, Edwin R. Miranda, Prabhakaran Kumar, Bellur S. Prabhakar, Jacob M. Haus

**Affiliations:** ^1^ Department of Kinesiology and Nutrition University of Illinois at Chicago Chicago Illinois; ^2^ Department of Kinesiology Iowa State University Ames Iowa; ^3^ Department of Microbiology and Immunology University of Illinois at Chicago Chicago Illinois; ^4^ School of Kinesiology University of Michigan Ann Arbor Michigan

**Keywords:** ADAM10, MyD88, postprandial, Toll‐like receptor 4

## Abstract

A high‐fat diet can induce inflammation and metabolic diseases such as diabetes and atherosclerosis. The receptor for advanced glycation endproducts (RAGE) plays a critical role in metabolic disease pathophysiology and the soluble form of the receptor (sRAGE) can mitigate these effects. However, little is known about RAGE in the postprandial condition and the effect of exercise in this context. Thus, we aimed to determine the effects of a single high‐fat meal (HFM) with and without prior exercise on peripheral blood mononuclear cell (PBMC) RAGE biology. Healthy males (*n *=* *12) consumed a HFM on two occasions, one without prior exercise and one 16–18 hours following acute aerobic exercise. Total soluble RAGE (sRAGE) and endogenous secretory RAGE (esRAGE) were determined via ELISA and cleaved RAGE (cRAGE) was calculated as the difference between the two. Isolated PBMCs were analyzed for RAGE, ADAM10, TLR4, and MyD88 protein expression and ADAM10 activity. The HFM significantly (*P *<* *0.01) attenuated sRAGE, esRAGE, and cRAGE by 9.7%, 6.9%, and 10.5%, respectively. Whereas, the HFM increased PBMC RAGE protein expression by 10.3% (*P *<* *0.01), there was no meal effect on PBMC TLR4, MYD88, or ADAM10 protein expression, nor ADAM10 activity. There was also no exercise effect on any experimental outcomes. These findings suggest that PBMC RAGE and soluble RAGE may be important in the postprandial response to a HFM, and that prior aerobic exercise does not alter these processes in young healthy adult males. The mechanisms by which a HFM induces RAGE expression and reduces circulating soluble RAGE isoforms requires further study.

## Introduction

Western dietary patterns include the frequent intake of large, high‐fat, energy‐dense meals. Consumption of a single high‐fat meal (HFM) increases postprandial blood lipid accumulation (Fuller et al. 2017) and induces cellular dysregulation through transient oxidative stress and inflammation (Erridge et al. 2007). When consumed chronically, a high‐fat diet stimulates chronic low‐grade inflammation in metabolically active tissues such as skeletal muscle and adipose tissue, which leads to obesity‐related diseases (Grimble 2002). Specifically, chronic low‐grade inflammation plays a critical role in the development of type 2 diabetes mellitus (T2DM) (Stehouwer et al. 2002), atherosclerosis (Okazaki et al. 2014), and metabolic syndrome (Nishimura et al. 2009).

Regular exercise training decreases metabolic disease risk (Bassuk and Manson 2005; Bakker et al. 2017), improves disease‐related complications (Hu et al. 2001), and decreases mortality rates (Blair et al. 1989; Wei et al. 2000). More specifically, chronic exercise improves postprandial lipemia (Cohen et al. 1989; Merrill et al. 1989), inflammation measured via inflammatory cytokines (Panagiotakos et al. 2005; Dixon et al. 2009), and oxidative stress (Miyazaki et al. 2001; Rector et al. 2007; Jenkins et al. 2011). Despite these well‐known effects of regular exercise, less is known about the ability of a single bout of exercise to protect against metabolic disease. We and others have shown metabolic improvements with a single bout of exercise (Tsetsonis et al. 1997; Fuller et al. 2017); however, results remain inconsistent and vary heavily based on study population, timing, and mode of exercise. Of note, aerobic exercise performed 16–18 hours prior to a HFM has readily shown robust protection during the postprandial period (Tsetsonis et al. 1997; Petitt et al. 2003). Altogether it remains unclear whether acute exercise is a successful strategy in preventing acute metabolic dysfunction following a HFM and what cellular mechanisms may be involved.

The receptor for advanced glycation endproducts (RAGE) is a multiligand transmembrane receptor known to play an integral role in mediating disease pathophysiology. Current knowledge indicates that in healthy phenotypes, RAGE is highly expressed in lung tissue but lowly expressed in other tissues (Brett et al. 1993). However, under pathologic conditions such as T2DM and diabetic nephropathy (Tanji et al. 2000), and Alzheimer's Disease (Miller et al. 2008), RAGE expression is greatly increased. Once activated by ligand binding, RAGE initiates many downstream signaling pathways that converge on the transcription factor nuclear factor‐kappa B (NF‐*κ*B) (Schmidt et al. 2001). NF‐*κ*B upregulates transcription of its target genes which include inflammatory cytokines, adhesion molecules, and RAGE itself (Ott et al. 2014). This induction of RAGE by NF‐*κ*B further amplifies the initial signal, which is how RAGE plays a key role in converting an acute proinflammatory state to chronic cellular dysfunction (Schmidt et al. 2001; Bierhaus et al. 2005). In addition, RAGE also directly induces the formation of reactive oxygen species through NADPH oxidases (Wautier et al. 2001). These inflammatory and oxidative stress producing mechanisms implicate RAGE as a key player in disease pathophysiology.

Specific to diet‐induced inflammation, plasma levels of RAGE ligands increase with high saturated‐fat diets (Lopez‐Moreno et al. 2017) and in mice, RAGE is critical in mediating inflammation and insulin resistance in response to high‐fat feeding (Song et al. 2014). Toll‐like receptors (TLRs) are also involved in mediating diet‐induced inflammation as free fatty acids (FFAs) are specific activators of Toll‐like receptor 4 (TLR4) in macrophages (Huang et al. 2012). Upon binding, FFAs activate TLR4, which then induces NF‐*κ*B, activates c‐Jun N‐terminal kinase (JNK), and recruits myeloid differentiation primary response 88 (MyD88) (Shi et al. 2006). TLR4 and the aforementioned downstream events lead to the release of inflammatory cytokines and are also partly required for HFD‐induced inflammation in mice (Shi et al. 2006). RAGE and TLR4 have a shared signaling pathway that converges on MyD88 and is involved in the release of inflammatory cytokines (Sakaguchi et al. 2011; Nielsen et al. 2017).

A circulating form of RAGE, termed soluble RAGE (sRAGE), acts as a ligand decoy and attenuates RAGE signaling (Zeng et al. 2004). There are two isoforms of sRAGE: cleaved RAGE (cRAGE) and endogenous secretory RAGE (esRAGE). While produced via unique mechanisms, both isoforms lack the transmembrane domain required for signaling and function as competitive inhibitors to RAGE. cRAGE results from proteolytic cleavage of the extracellular domain of membrane‐bound RAGE by A Disintegrin and metalloproteinase domain‐containing protein 10 (ADAM10) (Raucci et al. 2008), whereas alternate splicing of the RAGE pre‐mRNA produces esRAGE (Yonekura et al. 2003). Furthermore, the cRAGE:esRAGE ratio can be used to determine the relationship between these independently produced isoforms and can lend insight into their unique modulation. Individuals with conditions related to chronic low‐grade inflammation, such as obesity and T2DM, present with diminished sRAGE profiles (Dozio et al. 2016; Miranda et al. 2017a) and greater RAGE protein expression (Subramanian et al. 2017).

RAGE is highly expressed in many tissues, including peripheral blood mononuclear cells (PBMCs) (Sourris et al. 2010). PBMCs provide a strong platform for investigating postprandial inflammation in the context of exercise and disease because they are sensitive to both dietary manipulations (Wan et al. 2014) and exercise interventions (Jenkins et al. 2011). For example, a diet high in saturated fats upregulates PBMC RAGE mRNA expression (Lopez‐Moreno et al. 2016) and we previously demonstrated that an acute bout of endurance exercise attenuates phosphorylation of PBMC NF‐*κ*B (Fuller et al. 2017). Furthermore, PBMC inflammation and macrophage infiltration are implicated in the development of insulin resistance, diabetes, and atherosclerosis (McArdle et al. 2013).

Despite the well‐established role of RAGE in inflammatory signaling, little is known about PBMC RAGE and sRAGE in the postprandial period following a HFM. Further, it has yet to be determined if prior exercise mediates postprandial sRAGE and PBMC RAGE. Therefore, the aim of this project was to determine the effects of an acute HFM, with or without prior moderate aerobic exercise, on PBMC RAGE biology. We investigated plasma sRAGE isoforms, PBMC RAGE, ADAM10, TLR4, and MyD88 protein expression and PBMC ADAM10 activity. We hypothesized that the HFM would decrease sRAGE and that this decrease would be related to increased PBMC protein expression of RAGE, TLR4, and MyD88 and decreased PBMC ADAM10 activity and expression. Furthermore, we hypothesized that prior aerobic exercise would be protective against the postprandial decrease in sRAGE and increase in PBMC RAGE following the HFM.

## Materials and Methods

### Experimental protocol and participants

The purpose of this study was to investigate RAGE responses to an acute HFM. Demographic and experimental data from the main cohort have been previously published (Fuller et al. 2017), however, this is the first report of RAGE, sRAGE, and related outcomes in these subjects. A second validation cohort was recruited to investigate RAGE expression across immune cell populations via flow cytometry. All participants were nonsmokers and free from known metabolic or immunologic diseases.

Participants in the main cohort (*n *=* *12) provided written informed consent prior to enrollment, and procedures were completed in accordance with the Iowa State University Institutional Review Board. The full experimental procedures have been outlined previously (Fuller et al. 2017). In brief, healthy male participants ages 18–35 reported to the laboratory on four separate occasions. Visit 1 consisted of metabolic and anthropometric measurements which included a body composition and aerobic fitness (VO_2_ peak) assessment (Fuller et al. 2017). The two HFM visits (one control HFM visit and one HFM visit with prior aerobic exercise) were completed in a randomized crossover design. With the exception of the exercise visit (which occurred the afternoon prior to one of the HFM visits), all study visits were separated by 4–7 days.

For the prior exercise HFM visit, participants cycled on a cycle ergometer for 45mins at 65% VO_2_ peak. The acute exercise visit took place in the afternoon, 16–18 hours prior to the morning meal. Participants consumed an evening meal between the exercise and HFM visits, recorded their dietary intake for 3 days prior to each HFM, and were instructed to match their intake for each trial. For each of the HFM visits, participants reported to the laboratory following a 12–14 hours fast. Participants were then instructed to consume the HFM in less than 10mins, and time of consumption was matched between the two trials. The HFM consisted of two commercially available sausage, egg, and cheese breakfast sandwiches and an 8 oz glass of chocolate milk. This meal totaled to 1000 kcals and was 57% fat. Full details of the meal composition were reported previously (Fuller et al. 2017). Physical activity was tracked and matched prior to the two HFM visits and all participants refrained from purposeful physical activity 24 hours before each trial.

A separate validation cohort of participants (*n *=* *5; Age = 28 ± 2 years; BMI = 28.7 ± 2.6 kg/m^2^; VO_2_ Peak = 43.1 ± 4.8 mL/kg/min, 3.6 ± 0.4 L/min) was recruited to confirm RAGE expression across different circulating immune cell populations using flow cytometry (described below). Similar to the primary cohort, subjects were ages 18–35 and free from any known metabolic or immunologic diseases. By design, anthropometric and metabolic characteristics were similar between the two cohorts. Study procedures for the validation cohort were approved by University of Illinois at Chicago's Institutional Review Board and participants provided written informed consent prior to enrollment.

### Plasma metabolites

Prior to consumption of the HFM, an indwelling antecubital vein catheter was inserted for serial blood sampling. After baseline sampling, postprandial draws were obtained at 30 minutes, 1, 2, 3, and 4 hours following the HFM. Plasma glucose, insulin, and nonesterified fatty acids (NEFAs) were quantified at all time points, whereas plasma sRAGE and esRAGE were measured at baseline and 4 hours post‐HFM. The 4 hours postprandial sampling was modeled from previous studies that showed an effect of different dietary interventions on postprandial AGEs and PBMC RAGE (Lopez‐Moreno et al. 2016, 2017). Glucose (Sigma‐Aldrich, St. Louis, MO; Inter‐Assay CV = 3.9%, Intra‐Assay CV = 2.6–4.5%) and NEFAs (Wako Diagnostics, Richmond, VA; Inter‐Assay CV = 2.9%, Intra‐Assay CV = 6.6–18.1%) were measured via enzymatic assays. Insulin (Crystal Chem, Elk Grove, IL; Inter‐Assay CV = 4.7%, Intra‐Assay CV = 3.3–7.0%), sRAGE (R&D, Minneapolis, MN; Inter‐Assay CV = 2.5%, Intra‐Assay CV = 2.2–2.8%), and esRAGE (As One International, Santa Clara, CA; Inter‐Assay CV = 2.4%, Intra‐Assay CV = 2.0–2.6%) were all measured via commercially available ELISAs. As previously described (Miranda et al. 2017a,b), the cRAGE isoform was calculated by subtracting esRAGE from total sRAGE and the cRAGE:esRAGE ratio was calculated thereafter. Homeostatic model assessment of insulin resistance (HOMA‐IR) was calculated as the product of fasting plasma insulin and fasting plasma glucose divided by 22.5.

### PBMC isolation and analysis

Ficoll Paque Plus (Sigma‐Aldrich, St. Louis, MO) was used to isolate PBMCs from whole blood at baseline and 4 hours post‐HFM. PBMCs were stored in cell lysis buffer with phosphatase and protease inhibitor cocktails (Cell Signaling, Danvers, MA) at −80°C until further analysis. Protein concentration was measured via BCA assay (Thermo Scientific, Waltham, MA).

Equal protein within each participant, averaging 11ug of total protein, was loaded and run on 8–16% polyacrylamide gradient gels (Bio‐Rad, Hercules, CA). Protein was transferred to nitrocellulose membranes and blocked with Odyssey TBS Blocking Buffer (Li‐Cor, Lincoln, NE). Membranes were incubated in primary antibody at 4°C overnight and following washing, incubated in secondary antibody for 1 hour at room temperature. Primary antibodies were incubated at the following concentrations: RAGE (Abcam ab3611, Cambridge, MA): 1:1000, ADAM10 (Cell Signaling 14194S): 1:250, TLR4 (Santa Cruz Biotechnology sc‐293072, Dallas, TX): 1:1000, MyD88 (Abcam ab133739): 1:500, GAPDH (Cell Signaling 14C10): 1:5000. Secondary antibody (Li‐Cor) for the appropriate species was applied at 1:20,000 and membranes were imaged using near infrared fluorescence (Li‐Cor). All proteins of interest were normalized to GAPDH and the fold change from baseline during the control HFM visit was calculated for each participant.

PBMC ADAM10 activity was assessed using a fluorometric assay (AnaSpec, Fremont, CA; Intra‐Assay CV = 5.0%). For each baseline and 4 hours post‐HFM sample, 10 micrograms of protein were loaded onto the plate in duplicate. All ADAM10 activity readings were made relative to the pre‐HFM control visit value and reported as relative fluorescence units (RFU).

### Flow cytometry

From only the validation cohort, circulating immune cells were isolated via red blood cell lysis solution for analysis via flow cytometry. Cells were isolated in the morning following a 12 hours fast. Following isolation, cells were washed twice with staining buffer (0.5% BSA). To probe for RAGE expression, cells were incubated in primary antibody (Abcam ab89911) at 1:200 for 1 hour at 4°C. Following primary incubation, cells were incubated in 1:1600 secondary antibody (Abcam ab150113) for 1 hour at 4°C. Finally, cells were surface stained at 1:200 for 1 hour at 4°C with anti‐CD3‐eFluor‐450 (Thermo Fisher 48‐0037‐42), anti‐CD14‐APC (Thermo Fisher 17‐0149‐42), and anti‐CD16‐PE‐Cy7 (BD Bioscience 560,716, Franklin Lakes, NJ) to distinguish populations of lymphocytes, monocytes, and neutrophils, respectively. Cells were washed twice with staining buffer between each stain cycle. Cells were analyzed by CyAn ADP Analyzer (Beckman and Coulter) and data were analyzed using Summit v4.3 software (Beckman and Coulter).

### Statistics

All statistical analyses were run using SPSS Statistics 23 (IBM, Armonk, NY) and data are expressed as mean ± SEM. All variables were checked for normal distribution using the Shapiro‐Wilk normality test. A two‐way repeated measures ANOVA was used to assess differences with the HFM and exercise and the Bonferroni post hoc analysis was applied when appropriate. Pearson's correlation was used to determine relationships between RAGE‐related PBMC proteins and blood metabolites. *P* < 0.05 was considered statistically significant.

## Results

### Participant characteristics

Participant characteristics for the main cohort have been previously reported (Fuller et al. 2017) and are represented in Table 1. Fasting HOMA‐IR was not different between the two visits (data not shown) and there were no significant differences in age, BMI, or VO_2_ peak between the main cohort and the validation cohort.

**Table 1:** Study population characteristics

Variable (units)	Mean ± SEM
*N*	12
Age (year)	23 ± 1
BMI (kg/m^2^)	24.2 ± 1.1
% Body fat	18.6 ± 2.1
Fasting insulin (mU/L)	5.5 ± 1.2
Fasting glucose (mmol/L)	5.9 ± 0.2
HOMA‐IR (AU)	1.5 ± 0.3
VO_2_ peak (ml/kg/min)	44.7 ± 2.5
VO_2_ peak (L/min)	3.4 ± 0.2

Participant characteristics have been previously reported (Fuller et al. 2017). BMI, body mass index; HOMA‐IR, Homeostatic Model Assessment of Insulin Resistance; VO_2_ peak, volume of oxygen uptake.

### The effect of an acute HFM on plasma sRAGE

**Figure 1:**
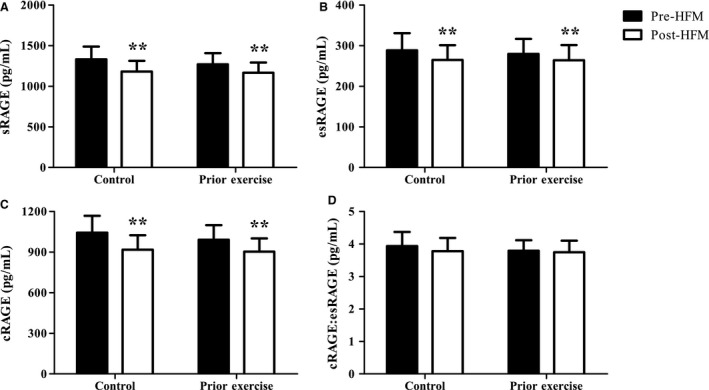
Decreased postprandial total sRAGE, esRAGE, and cRAGE. Changes in total sRAGE (A), esRAGE (B), cRAGE (C), and the cRAGE:esRAGE ratio (D) with an acute HFM and prior aerobic exercise. Data were analyzed via two‐way ANOVA and data are presented as mean ± SEM. *n *=* *12 for all sRAGE isoforms. **Main effect of HFM *P *<* *0.01.

The acute HFM decreased (*P *<* *0.01) plasma total sRAGE, esRAGE, and cRAGE (Fig. 1). Since esRAGE and cRAGE are produced by independent mechanisms, we calculated a ratio of these products to gain insight into their relative contribution to one another. However, there was no change in the cRAGE:esRAGE ratio, nor was there an effect of prior aerobic exercise on any of the sRAGE variables. To investigate the potential mechanisms behind the attenuation of sRAGE, we calculated the role that each isoform played in the overall decrease in total sRAGE. From this, we determined that cRAGE accounted for 73 ± 12% of the total sRAGE change, whereas esRAGE drove the remaining 27 ± 12%.

### Postprandial PBMC ADAM10 activity

Having identified cRAGE as the main driver in the postprandial sRAGE response, we investigated mechanisms of cRAGE production. Given that ADAM10 is the primary metalloproteinase responsible for RAGE ectodomain cleavage (Metz et al. 2012), we assayed ADAM10 activity in PBMC homogenates. There was no effect of the meal (Fold change: Pre‐HFM = 1.03 ± 0.04 RFU, Post‐HFM = 1.08 ± 0.06 RFU) or exercise (Fold change: Control = 1.09 ± 0.07 RFU, Prior Exercise = 1.02 ± 0.03 RFU) on PBMC ADAM10 activity (*P *=* *0.24 and 0.25 respectively). Furthermore, no correlations were found between PBMC ADAM10 activity and any of the sRAGE variables or PBMC protein targets of interest.

### Altered PBMC protein expression with acute high‐fat feeding

**Figure 2:**
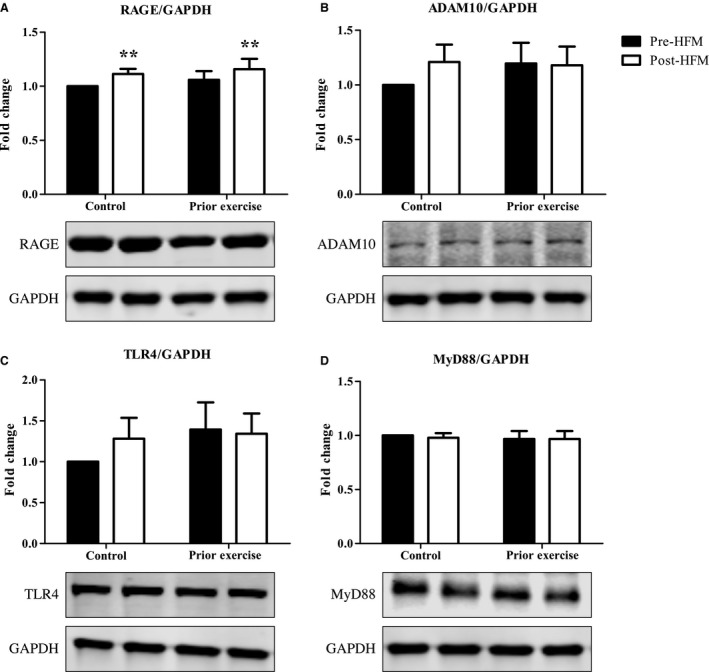
Altered PBMC Protein Expression with acute high‐fat feeding. Normalized and fold change results show PBMC RAGE (A), ADAM10 (B), TLR4 (C), and MyD88 (D) with acute high‐fat feeding with and without prior aerobic exercise. Western blot bands follow the same order as the figure bars. Data were analyzed via two‐way ANOVA and data are presented as mean ± SEM. *n* = 12 for all proteins represented. ***Main effect of HFM *P* = 0.001.

We assessed PBMC protein expression of RAGE, ADAM10, TLR4, and MyD88, all of which play a role in RAGE signaling. There was a main effect of the HFM on PBMC RAGE, where RAGE protein expression was 11 ± 5% and 9 ± 4% higher 4 hours post‐HFM for the control and prior exercise meal visits, respectively (*P *=* *0.001, Fig. 2A). There was no effect of the meal on ADAM10, TLR4, or MyD88 PBMC protein expression. There were also no effects of prior exercise or any interactions between the HFM and exercise interventions on our PBMC protein targets of interest (Figs. 2B–D).

Despite no change in TLR4, we found that both the baseline levels and the 4 hours postprandial change in PBMC RAGE and TLR4 protein expression were positively correlated (*r *=* *0.60, *P *<* *0.01; *r *=* *0.55, *P *<* *0.01). TLR4 and MyD88 protein expression were also positively associated at baseline (*r *=* *0.54, *P *<* *0.01).

### Postprandial insulin and NEFA changes with a HFM

**Figure 3:**
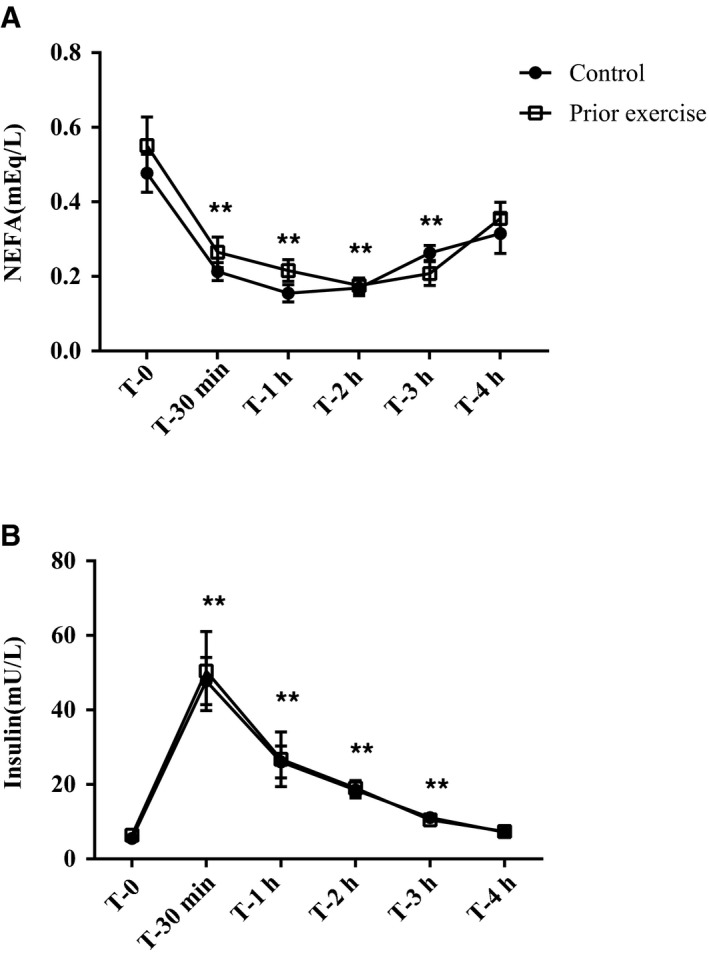
High‐fat meal‐induced rise in insulin and blunting effect on NEFAs. Baseline and postprandial plasma insulin (A) and plasma NEFA (B). Data are presented as mean ± SEM. Data were analyzed via a repeated measures ANOVA; *P < 0.05 compared to baseline, **P < 0.01 compared to baseline. *n* = 12 for all plasma metabolites.

As seen in Figure 3, there was a main effect of the HFM on both plasma insulin and NEFAs (*P *<* *0.001, *P *<* *0.001 respectively), whereby consumption of a single HFM increased plasma insulin and decreased plasma NEFAs. However, there was no effect of exercise on insulin or NEFAs and neither insulin nor NEFAs were related to sRAGE levels or RAGE‐related PBMC protein expression.

### RAGE expression varies among cell subpopulations

**Figure 4:**
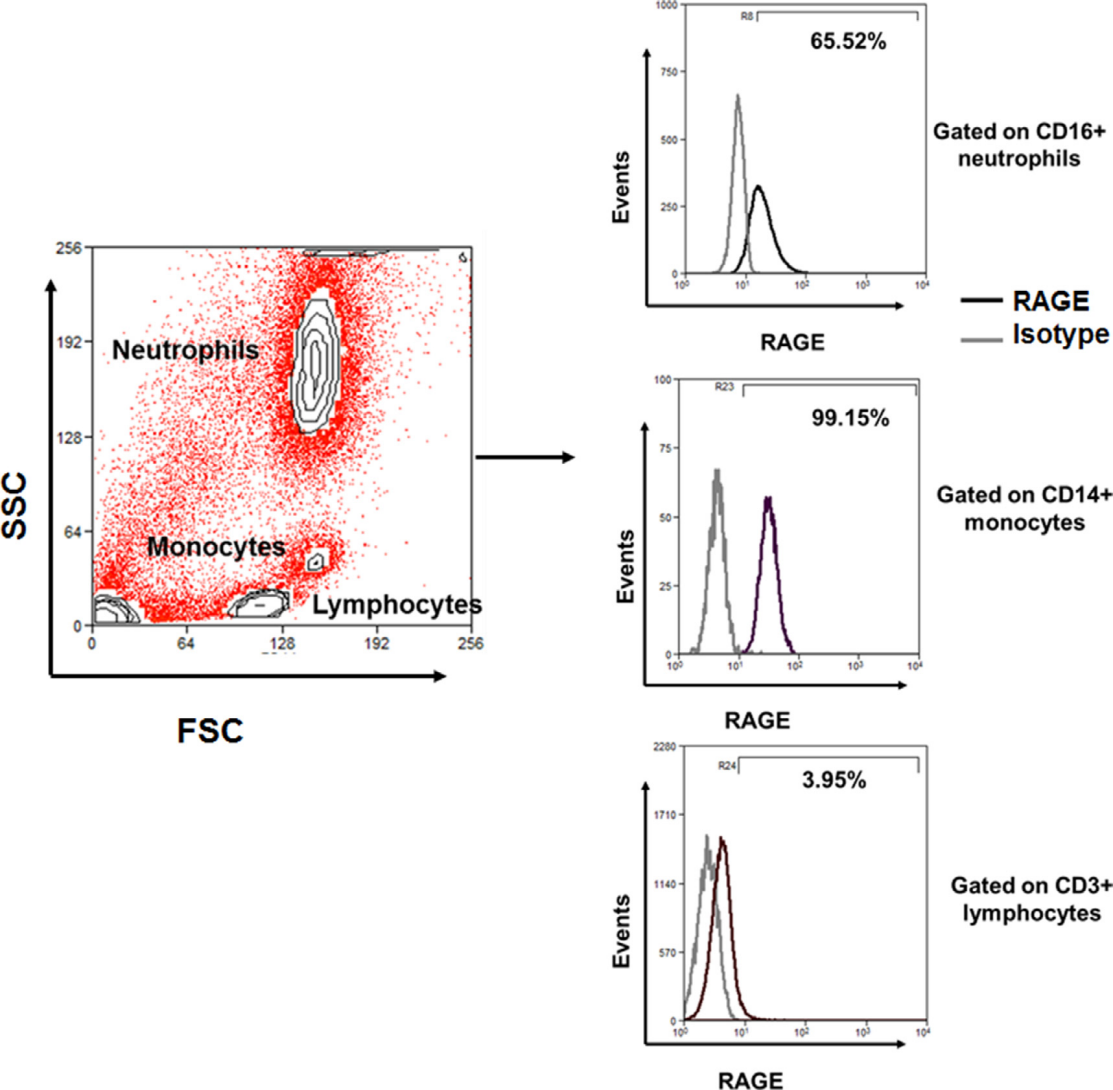
RAGE expression differs among circulating immune cell populations. Total leukocytes were isolated from peripheral blood were analyzed by flow cytometry. Neutrophils, monocytes, and T‐lymphocytes were gated based on their FSC versus SSC scatters followed by CD16, CD14, and CD3 expression respectively. Representative overlay histograms show percentages of RAGE cells within gated populations after background correction with isotype controls.

To further understand circulating immune cell expression of RAGE, we used flow cytometry to assess surface expression of RAGE in monocytes, neutrophils, and lymphocytes in a separate validation cohort (Fig. 4). Following a 12 hours fast, almost all (94 ± 3%) monocytes expressed RAGE, about half (45 ± 14%) of neutrophils were RAGE positive, and very few (1 ± 0%) lymphocytes expressed RAGE.

## Discussion

RAGE has long been associated with inflammation in metabolic disease and recent work has highlighted the protective role of sRAGE in attenuating RAGE signaling (Leclerc et al. 2007; Zong et al. 2010). In this study, we investigated the effect of an acute HFM, with and without prior exercise, on PBMC RAGE signaling and sRAGE. We found that a HFM increased PBMC RAGE protein expression while simultaneously decreasing plasma total sRAGE, esRAGE, and cRAGE. Additionally, the changes in PBMC RAGE and TLR4 protein expression with the HFM were correlated. Despite effects of the HFM, we did not observe changes in response to prior acute exercise on any of the experimental outcomes.

The increased expression of PBMC RAGE with an acute HFM is in line with previous reports of high‐fat diet‐induced PBMC RAGE mRNA in humans (Lopez‐Moreno et al. 2016) and liver RAGE expression in rodents (Cheng et al. 2017). Our findings, however, show that increased RAGE signaling can occur with one acute exposure to a HFM. While we did not measure inflammatory cytokines in this study, previous reporting of HFM‐induced inflammation (Erridge et al. 2007) would suggest these effects may be mediated by RAGE. Certainly, future studies should investigate whether HFM‐induced PBMC RAGE expression directly results in the release of inflammatory mediators and establish the importance of RAGE in acute postprandial inflammation or the development of chronic low‐grade inflammation with prolonged exposure to high‐fat feeding.

Keeping in mind the small sample size, the high surface expression of RAGE in monocytes suggest that these cells may drive the postprandial changes in PBMC RAGE signaling in healthy participants. AGE‐modified proteins have previously been shown to increase chemotactic activity for monocytes prior to the formation of vascular lesions (Kirstein et al. 1990; Schmidt et al. 1993), and RAGE can mediate subsequent macrophage infiltration and atherosclerotic plaque formation (Cipollone et al. 2003). However, we cannot rule out the role of activated T cells in mediating RAGE responses to a HFM. Other reports in healthy participants reveal similar basal levels of RAGE and suggest that T‐cell activation is required for CD4+ and CD8+ RAGE expression to exceed 5% (Akirav et al. 2012). Thus, it is possible that T‐cell RAGE may play a larger role in postprandial changes in a population with obesity, diabetes, or atherosclerosis, as disease‐related or high‐fat diet‐induced inflammation leads to the inappropriate activation of T cells and subsequent disease progression (Norata et al. 2015; Mauro et al. 2017).

We also report decreased total sRAGE, esRAGE, and cRAGE in response to the HFM, despite no differences in PBMC ADAM10 activity or protein expression. This contrasts with our hypothesis, as we expected to see decreased PBMC ADAM10 activity and protein expression as ADAM10 cleaves membrane‐bound RAGE to produce cRAGE (Raucci et al. 2008). It is possible that the decreased cRAGE and total sRAGE pools are in fact due to decreased ADAM10 activity, but that this phenomenon is occurring in peripheral tissues rather than PBMCs. Future work should investigate the effect of an acute HFM on skeletal muscle and adipose tissue ADAM10 to determine the source of diminished sRAGE. We chose not to explore mechanisms of esRAGE production as our total sRAGE change was being driven mostly by cRAGE. However, these alternate splicing events should also be investigated in response to a HFM to confirm if PBMCs play a role in regulating postprandial sRAGE production.

Contrary to our hypothesis, we did not find any evidence of NEFA‐driven increases in PBMC TLR4 protein expression with the HFM. This is likely due to insulin suppression of lipolysis in the postprandial period as well as an uptake of circulating NEFAs by peripheral tissues (Rebrin et al. 1996; Coppack et al. 1999). NEFAs begin to rise above baseline 5–6 hours following a meal (Bickerton et al. 2007) and therefore we may have seen a relationship between NEFAs and TLR4 had we continued sampling after the 4 hours time point. Despite no change in PBMC TLR4 or MyD88 expression with the HFM, our results support a relationship between RAGE, TLR4, and MyD88 in PBMCs. Not only did we see that baseline RAGE and MyD88 are related to baseline TLR4, but that the postprandial change in RAGE and TLR4 expression are also related. These data support a common signaling pathway between RAGE and TLR4/MyD88 which has independently been shown to result in the activation of NF‐*κ*B (van Beijnum et al. 2008). Whether NEFA‐mediated TLR4 activation with HFM plays a mechanistic role in the activation and/or expression of PBMC RAGE requires further investigation.

Acute, moderate intensity aerobic exercise did not attenuate postprandial RAGE signaling in PBMCs. Despite evidence suggesting an anti‐inflammatory effect of exercise (Starkie et al. 2003; Gleeson et al. 2011), several methodological differences, such as the timing (Ispirlidis et al. 2008; Chatzinikolaou et al. 2010), intensity (Ostrowski et al. 2000; Peake et al. 2005), and mode of exercise (Balducci et al. 2010) may explain the absence of an exercise effect. In addition, the lack of an exercise response may be due to the health of our participants. This study was completed with young, healthy men, and prior work suggests that individuals with T2DM experience a greater anti‐inflammatory response to exercise compared to obese or lean counterparts (Dekker et al. 2007). Though physical activity was matched in the days preceding each trial, it is possible that exercise performed 24–72 hours prior to the visit could be confounding our results. The application of these findings is limited by our study population and study design. Future work should investigate these relationships across sex, age, and disease status. Furthermore, a control meal should be utilized in the future to isolate the effects of a HFM from that of a meal alone.

Our study has shown that an acute HFM attenuates postprandial sRAGE, an important competitive inhibitor of RAGE. We also report HFM‐induced PBMC RAGE expression, which is likely driven by the monocyte population. Given no change in ADAM10, our data suggest that the postprandial decreases in sRAGE may be occurring through different mechanisms in PBMCs or modulation in tissues sensitive to postprandial metabolism. Future work should aim to determine the site of postprandial sRAGE regulation and if these acute metabolic changes contribute to the pathophysiology of chronic metabolic diseases.

## Conflict of Interest

None declared.
